# Hybrid CoO Nanowires Coated with Uniform Polypyrrole Nanolayers for High-Performance Energy Storage Devices

**DOI:** 10.3390/nano9040586

**Published:** 2019-04-09

**Authors:** Chunhai Yang, Hao Chen, Cao Guan

**Affiliations:** 1 School of Chemistry & Environment Engineering, Hubei University for Nationalities, Enshi 445000, China; yangchunhai001@163.com; 2 School of Engineering, Zhejiang A&F University, Hangzhou 311300, China; 3 Department of Materials Science and Engineering, National University of Singapore 117574, Singapore; 4 Institute of Flexible Electronics, Northwestern Polytechnical University, Xi’an 710072, China

**Keywords:** core-shell structure, conducting polymer coating, transition metal oxide, electrochemical capacitor, nanoarrays

## Abstract

Transition metal oxides with high theoretic capacities are promising materials as battery-type electrodes for hybrid supercapacitors, but their practical applications are limited by their poor electric conductivity and unsatisfied rate capability. In this work, a hybrid structure of CoO nanowires coated with conformal polypyrrole (Ppy) nanolayer is proposed, designed and fabricated on a flexible carbon substrate through a facile two-step method. In the first step, porous CoO nanowires are fabricated on flexible carbon substrate through a hydrothermal procedure combined with an annealing process. In the second step, a uniform nanolayer of Ppy is further coated on the surfaces of the CoO nanowires, resulting in a hybrid core-shell CoO@Ppy nanoarrays. The CoO@Ppy aligned on carbon support can be directly utilized as electrode material for hybrid supercapacitors. Since the conductive Ppy coating layer provides enhanced electric conductivity, the hybrid electrode demonstrates much higher capacity and superior rate capability than pure CoO nanowires. As a further demonstration, Ppy layer can also be realized on SnO_2_ nanowires. Such facile conductive-layer coating method can be also applied to other types of conducting polymers (as the shell) and metal oxide materials (as the core) for various energy-related applications.

## 1. Introduction

Recent years have witnessed the fast-growing requirements for clean energy and ever-growing costume demands are constantly driving research attentions to sustainable energy storage devices with better performance. As one of the most promising sustainable devices for energy storage, electrochemical capacitor (also named supercapacitor) can provide fast charge-discharge capability and high specific capacitance with long-time cycling stability; thus, it is quite favorable for the next-generation electrochemical energy storage devices [1,2,3,4,5]. Among the candidates for hybrid supercapacitors, carbonaceous materials, including carbon nanotubes, graphene and active carbon, could provide high electric conductivity with good chemical stability and stable cycling ability; however, they only store charges at their surfaces thus cannot provide high capacitance with high energy density [6,7,8,9]. As another type of electrode materials, transition metal oxides used as battery-type electrodes have much higher theoretical capacities based on redox reaction mechanism; however, their poor electric conductivity limit the wide applications of transition metal oxides [10,11,12,13,14,15,16].

Several strategies have been applied for the enhancement of the electrochemical performance of transition metal oxides, among which a promising way is the construction of nanostructured metal oxides with high specific surface areas, because the large surface-to-bulk ratio provides more sufficient sites for the effective electrochemical reaction between the electrode material and the electrolyte ions [17,18,19,20]. A second effective way is to directly fabricate nanoarrays of transition metal oxides on conductive current collectors. Such design can not only avoid the usage of unwanted nonconductive binders and additives, but also ensure the direct electrical and mechanical connection between the active electrode materials and the carbon substrate, which is essential for enhanced rate capability and mechanical stability [2,4,21]. The construction of hybrid electrode materials, such as core-shell structured and core-branched metal oxide-conducting polymers or metal oxide-carbonaceous materials is also a useful method to enhance the electrochemical properties of transition metal oxides, because the conductive polymers and carbon materials provide fast electron pathway for relatively insulated metal oxides, resulting in enhanced electric conductivity of the whole electrode. For instance, core-shell structured electrodes of MnO_2_@Ppy, MoS_2_/Polyaniline and SnO_2_@PEDOT have been fabricated and demonstrated to effectively integrate the advantages of each single component with enhanced electrochemical performance [22,23,24,25]. 

In this work, we intend to combine the above strategies into an integrated electrode. Herein, the structure design and facile fabrication of a core-shell CoO@Ppy material is demonstrated, where a nanolayer of Ppy is uniformly coated on the surfaces of porous CoO nanowires. In addition, the CoO@Ppy nanostructure is directly grown on flexible carbon support, which avoids the “dead mass” from the unwanted polymer binders and carbon additives. The thin and uniform Ppy shell layer also provides conductive path and surface protection for the inner porous CoO nanowire; thus, enhancing the fast electron transfer within the electrode integrity. With such structure design, the hybrid CoO@Ppy electrode demonstrated much higher capacity, preferable rate capability, as well as better cycling property than the pure CoO nanowires and Ppy nanowires. In addition, such conductive-layer coating method can be also extended to SnO_2_ nanowire, providing a promising nanostructure design tactics for enhanced electrochemical properties. 

## 2. Materials and Methods

### 2.1. Material Fabrication

The rational designed core-shell structured CoO@Ppy was synthesized through a facile two-step method. (1) The precursor for CoO was synthesized on carbon cloth by a hydrothermal-annealing process, based on our previous reports [26,27]. In detail, a homogeneous solution contains 2 mmol Co(NO_3_)_2_·6H_2_O, 4 mmol NH_4_F and 10 mmol urea with 50 mL deionized water was firstly obtained and then poured into a Teflon-lined stainless-steel autoclave, after which a carbon cloth substrate (with a typical size of 5 × 2 × 0.036 cm^3^, Cetech) was immersed into the obtained homogeneous reaction solution and reacted at 120 °C. After 8 h reaction, the carbon cloth coated with CoO precursor was cleaned with deionized water and annealed in Ar atmosphere for 2 h at 350 °C, after which porous CoO nanowires aligned on carbon cloth can be obtained [28]. (2) The Ppy nanolayer was further coated on the CoO nanowires by electrochemical deposition using a standard three-electrode system. The electrolyte was an aqueous solution (200 mL) containing 10 mM pyrrole and 50 mM Na_2_SO_4_. A saturated calomel electrode (SCE) was the reference electrode, and a piece of Pt plate (2 × 2 cm^2^) was the counter electrode. The electrochemical deposition of Ppy was performed by applying a current density of 1 mA for 30 min using an electrochemical workstation (Solarton 1470E, Solartron Analytical, Kingston, England).

### 2.2. Characterization

Scanning electron microscopy (SEM, Zeiss, Jena, Germany, 5.0 kV) and transmission electron microscopy (TEM, JEOL-2100F, JEOL, Tokyo, Japan) equipped with an energy dispersive X-ray spectrometer (EDX, JEOL, Tokyo, Japan) were used for the characterization of the samples. An AX/MX/UMX Balance (METTLER TOLEDO, maximum = 5.1 g; delta = 0.001 mg) was utilized to evaluate the masses of samples before and after the growth of materials. The mass loadings in our experiment are as follows: CoO 1.35–1.43 mg/cm^2^, CoO@Ppy 1.96–2.04 mg/cm^2^, Ppy 0.57–0.64 mg/cm^2^.

### 2.3. Electrochemical Measurement

Electrochemical tests were performed using a standard three-electrode electrochemical cell. CoO and CoO@Ppy were directly used as the working electrode, and no additional metal current collector was involved. A 2 M KOH was used as the electrolyte. A saturated calomel electrode (SCE) was used as the reference electrode, and a piece of Pt plate (2 × 2 cm^2^) was used as the counter electrode. All potentials measured were referred to the SCE electrode. In the electrochemical test, the specific capacity of the electrodes (mAh·g^−1^) and current density (A·g^−1^) and (mA·cm^−2^) were calculated based on the mass of the active materials (CoO and Ppy) and the geometric area of the electrodes, respectively. The specific capacity of the samples is calculated based on the following equation: C = It/m, where *t* is the discharge time, I is the applied discharge current and m is the mass of the active materials. For electrochemical impedance spectroscopy (EIS), the samples were tested at open circuit potential with a frequency range of 0.1 Hz to 100 kHz. 

## 3. Results and Discussion

### 3.1. Structural Characterization of the Electrode

**Figure 1:**
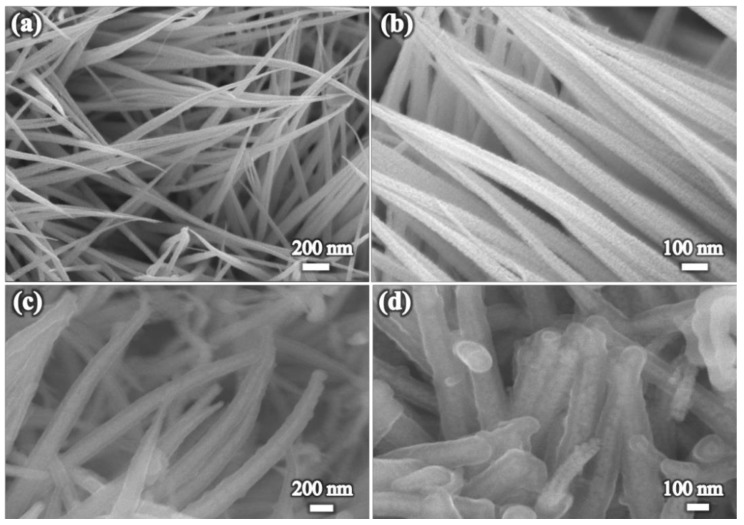
Scanning electron microscopy (SEM) images of (**a**,**b**) CoO nanowires on carbon cloth, (**c**,**d**) hybrid core-shell CoO@Ppy nanoarrays on carbon cloth.

Figure 1a,b displays the scanning electron microscope (SEM) images of the pure CoO nanowires aligned on flexible carbon support, from which one can see the nanowires are quite porous and the obtained CoO nanowires have typical diameters of around 100 nm with around 2 micrometers in length, which is in accordance with previous reported results [29,30]. After coating of Ppy layer by electrochemical deposition, the nanowire structure is well kept and becomes a little thicker (Figure 1c). From enlarged SEM image in Figure 1d, apparent core-shell structure can be seen as the two materials have different contrast under electron beam, and the CoO nanowire is uniformly coated by a uniform thin nanolayer. Such uniform growth of conducting polymer nanolayer on metal oxides is in accordance with previous works [31,32].

**Figure 2:**
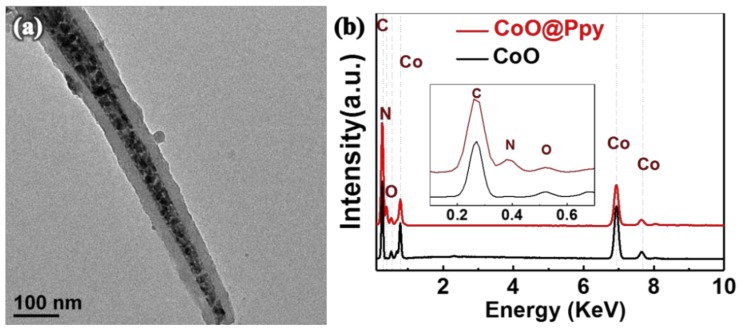
(**a**) Transmission electron microscopy (TEM) characterization and (**b**) energy dispersive X-ray spectrometer (EDX) results of core-shell CoO@Ppy nanoarrays.

The hybrid core-shell structure is further studied in the TEM experiment. Figure 2a shows the TEM result of a representative hybrid CoO@Ppy, which displays apparently that the porous CoO nanowire is coated by a uniform nanolayer with typical thickness of around 30 nm. EDX result (Figure 2b) obtained from CoO@Ppy also shows additional N peaks compared with that of bare CoO, further indicating the composite of CoO@Ppy is achieved [33]. To note, our work used electro- polymerization method for deposition of Ppy. As can be seen by SEM and TEM, the PPy layer is quite uniform, which is different from previously reported CoO@Ppy where the Ppy layer are not very uniform by using chemical polymerization process [33].

### 3.2. Electrochemical Properties of the Electrode

**Figure 3:**
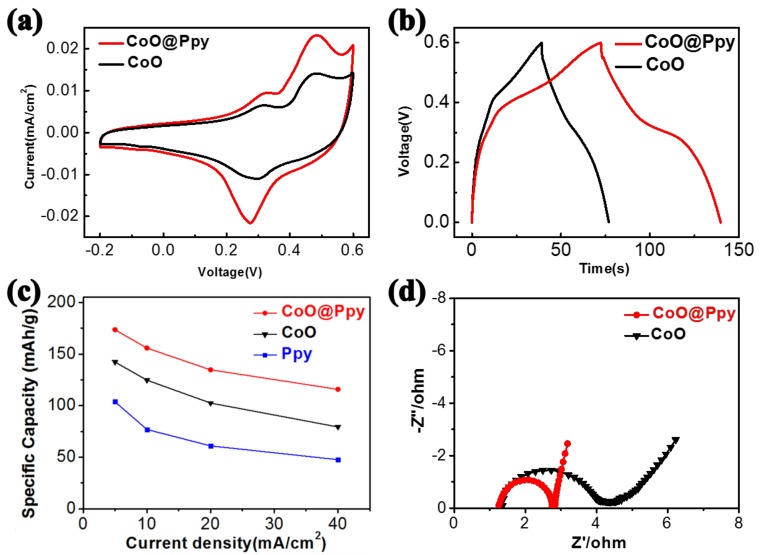
(**a**) CV curves, (**b**) charge-discharge curves, (**c**) rate capability, and (**d**) electrochemical impedance spectroscopy of the CoO nanowires and the core-shell CoO@Ppy nanoarrays.

To demonstrate the improvement of the surface Ppy coating, electrochemical performance of the CoO@Ppy and CoO were further measured using a three-electrode electrochemical test system. Figure 3a shows the CV curves of the pure CoO nanowires and hybrid CoO@Ppy nanowires. The CoO shows identical redox peaks in the CV test, indicating its battery-type behavior and suggesting the redox reaction between Co^2+^ and OH^−^. Such reactions can be illustrated as: CoO + OH^−^↔CoOOH + e^−^ and CoOOH + OH^−^↔CoO_2_ + H_2_O + e^−^, which is in accordance to the reported results on CoO electrode in alkaline electrolyte [26,33,34]. After being coated with a conductive Ppy shell layer, in the CV curve, the enclosed area that represents the capacity is largely increased, indicating that increased electrochemical activity with much higher capacity has been obtained. 

Figure 3b shows the galvanic charge-discharge test results, which further confirms the enhancement after Ppy coating. The hybrid core-shell structure displays a discharge capacity of 155.8 mAh·g^−1^ at a current density of 10 mA·cm^−2^, which is much larger than that for bare Ppy (142.2 mAh·g^−1^) at a same current density (Figure S2), showing the Ppy nanolayer coating results in much increased capacity.

The galvanic charge-discharge curves obtained from the two samples (CoO and CoO@Ppy) at various current densities of 5 to 40 mA·cm^−2^ are shown in Figure S1a,b. The current-capacity curves of the CoO@Ppy obtained is illustrated in Figure 3c. The capacities obtained from CoO@Ppy are always higher than the values of bare CoO when the current density increased from 5 to 40 mA·cm^−2^, indicating the electrochemical performance was enhanced after the conductive Ppy layer coating. In addition, the hybrid core-shell structure can maintain 66.7% of initial capacity when the discharge current density increased by eight times (from 5 to 40 mA·cm^−2^), while the number obtained from CoO is 55.8%, showing the core-shell structure of CoO@Ppy is more suitable for fast electrochemical reaction even at high current density. The above results confirm the enhanced rate capability after the Ppy coating. Noting that bare Ppy electrodeposited on carbon cloth (Figure S2) shows much smaller capacity (76.9 mAh·g^−1^) compared with 155.8 mAh·g^−1^ for CoO@Ppy, further confirming the synergistic effect by the combination of CoO and Ppy.

Electrochemical impedance spectroscopy (EIS), a useful data to represent the transport properties of an electrochemical system, was further performed to compare the two electrode materials (Figure 3d). The semicircle obtained in the high frequency region corresponds to the charge-transfer resistance occurred at the electrode/electrolyte interface. The CoO@Ppy shows a smaller radius than that for pure CoO, which indicates much enhanced electronic conductivity [35,36]. In addition, in the low frequency region, the CoO@Ppy also shows a more straight line along the imaginary axis, indicating the core-shell electrode has lower ion-diffusion resistance for fast reaction [25,37]. The above EIS results further shows that both charge transfer resistance and ion-diffusion resistance have been reduced after the Ppy coating, which accounts for much enhanced electronic conductivity and rate capability.

**Figure 4:**
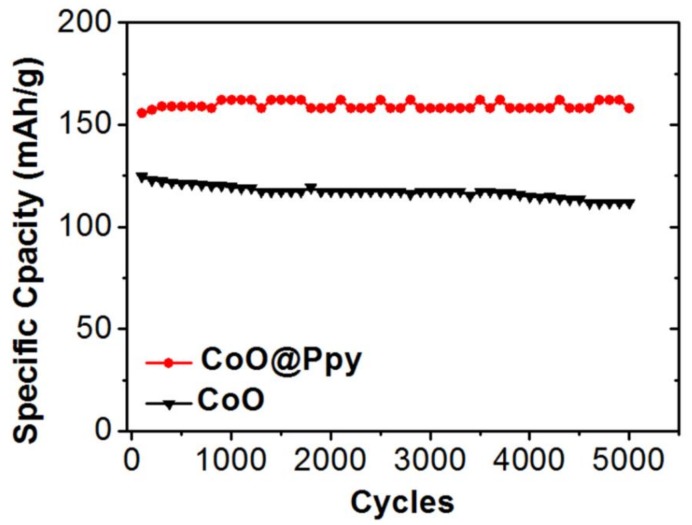
Cycling stability test of the CoO nanowires and the core-shell CoO@Ppy nanoarrays.

As another key parameter to evaluate electrode materials for hybrid supercapacitors, the cycling stability test was further carried out. As demonstrated in Figure 4, at a current density of 10 mA·cm^−2^ bare CoO nanowires shows a relative good cycling stability that after 5000 cycles it can well maintain 89 % of the initial capacity. After been coated with a conductive and protective layer of Ppy, the hybrid core-shell CoO@Ppy electrode delivers a much better cycling stability that the capacity is almost unchanged after 5000 cycles. 

Here, the reasons for the improvement of the hybrid CoO@Ppy electrode in electrochemical test can be originated from the following points: Firstly, the conductive Ppy nanolayer provides an electron “highway” that can ensure fast electron transport for the inside porous CoO thus ensure better rate capability. The above enhancement has also been confirmed by EIS results. Secondly, the porous structure of CoO and the thin nanolayer of Ppy can shorten the ion-diffusion length for the hybrid structure, which is confirmed by the EIS measurement. If the CoO is solid or the Ppy layer is too thick, the reaction between CoO and OH^−^ will be retarded with worse electrochemical properties. Lastly, the hybrid CoO@Ppy structures have direct electric and mechanical connections with the conductive carbon cloth substrate, where no binders or additives are involved, which also ensures good electrochemical performance. We believe the proper control of the length/diameter and porosity of CoO and the thickness of Ppy nanolayer can result in further optimized electrochemical performance, which will be conducted in the next step.

It is worth to mention that the above strategy of coating conductive layer on transition metal oxide can be also applied to other transition metal oxides and conductive materials. As a further demonstration, hybrid core-shell nanoarrays of SnO_2_ nanowires coating with Ppy thin layers have been achieved, as shown in Figure S3. The construction of core-shell metal oxide-conductive polymer hybrid materials would be a promising way for enhanced electrochemical properties and be applied in different energy-related areas.

## 4. Conclusions

To conclude, we reported a hybrid core-shell CoO@Ppy nanoarray obtained through a facile two-step process. The hybrid core-shell electrode with porous CoO core and conductive thin shell of Ppy demonstrated higher capacity and superior rate performance than the pure CoO nanowires. The out shell can also serve as a protection layer that maintains the long-term cycling ability of the core-shell electrode. Such hybrid core-shell structure design has been demonstrated as a promising way to enhance the performance of metal oxide and conducting polymer, which would provide a promising way for the improvement of the electrochemical performance of transition metal oxides, and additional improvement can be expected by optimizing the shell materials and thickness.

## Supplementary Materials

The following are available online at https://www.mdpi.com/2079-4991/9/4/586/s1, Figure S1: Charge-discharge curves of the samples, Figure S2: Characterization of Ppy deposited on carbon cloth, Figure S3: Characterization of SnO2@Ppy.
