# Supplementary material for: Hybrid CoO Nanowires Coated with Uniform Polypyrrole Nanolayers for High-Performance Energy Storage Devices
Source: Nanomaterials (Basel). 2019 Apr 9;9(4):586. doi: 10.3390/nano9040586 (PMC6523395; doi:10.3390/nano9040586)
Supplement: Supplementary file 1 [file nanomaterials-09-00586-s001.pdf]

Supplementary materials

Hybrid CoO Nanowires Coated with Uniform Polypyrrole Nanolayers for High-Performance Energy Storage Devices

Chunhai Yang [1], Hao Chen [2,3,*] and Cao Guan [4,*]

[1] School of Chemistry & Environment Engineering, Hubei University for Nationalities, Enshi 445000, China; yangchunhai001@163.com

[2] School of Engineering, Zhejiang A&F University, Hangzhou 311300, China

[3] Department of Materials Science and Engineering, National University of Singapore 117574, Singapore

[4] Institute of Flexible Electronics, Northwestern Polytechnical University, Xi'an 710072, China

* Correspondence: haochen@zafu.edu.cn (H.C.); iamcguan@nwpu.edu.cn (C.G.); Tel: +0571-63732700 (H.C.); +029-88492151 (C.G.)

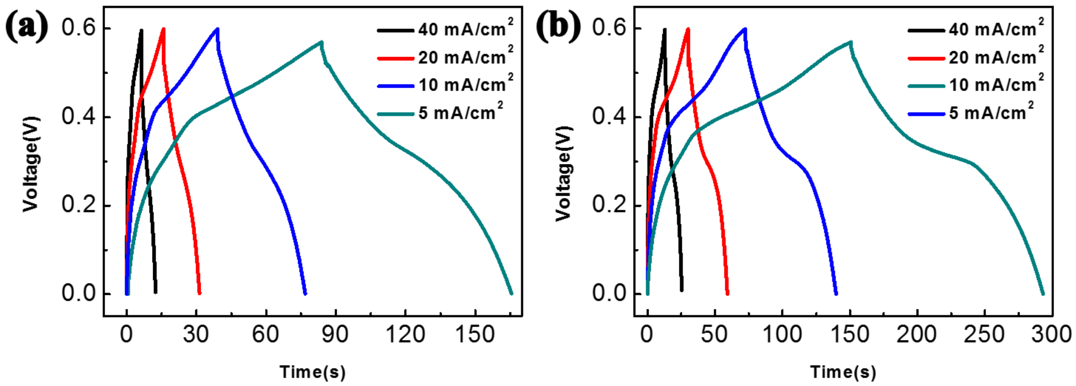

Figure S1. Charge-discharge curves of the samples. (a) Charge-discharge curves of CoO nanowires; (b) Charge-discharge curves of hybrid core-shell CoO@Ppy nanoarrays.

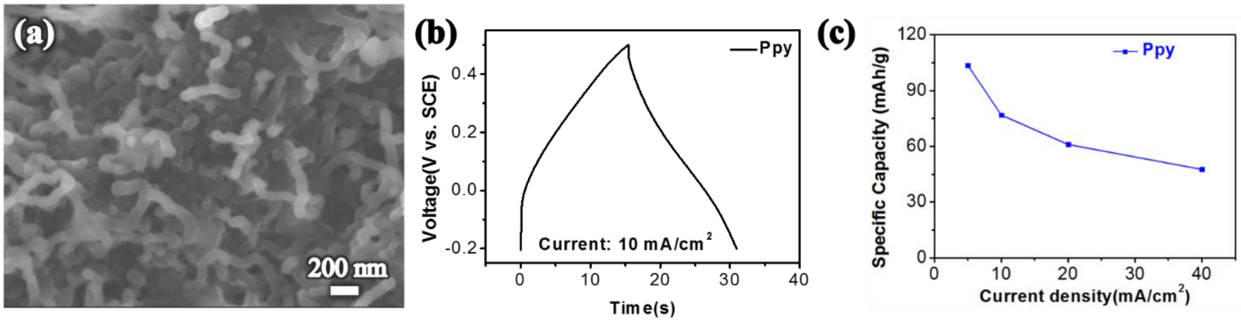

Figure S2. Characterization of Ppy deposited on carbon cloth. (a) SEM image of Ppy deposited on carbon cloth; (b) Charge-discharge curve and (c) rate performance of Ppy.

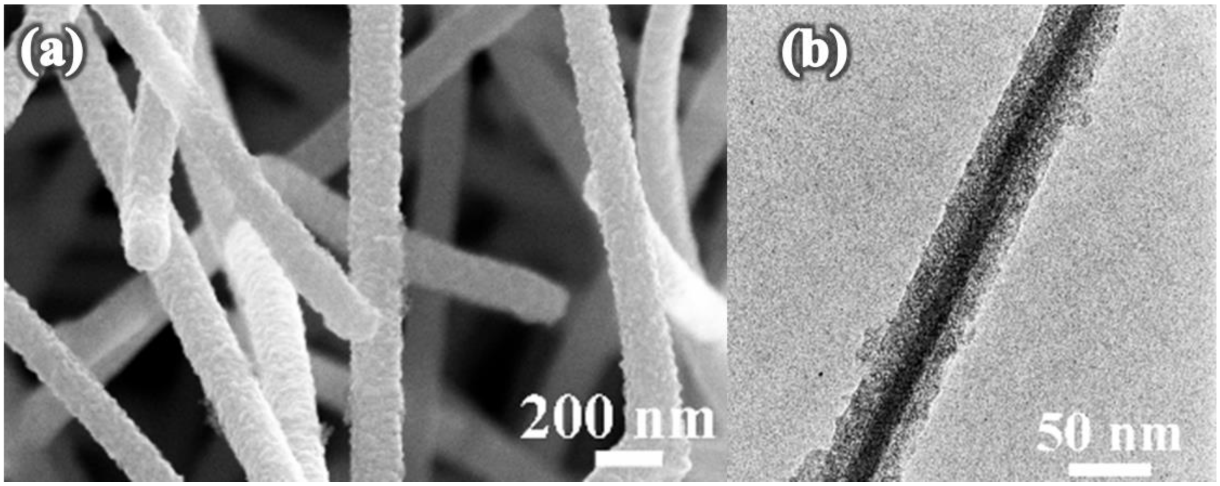

Figure S3. Characterization of SnO_2@Ppy. (a) SEM image and (b) TEM image of core-shell SnO_2@Ppy.
